# Knowledge and Awareness of Tourette’s Syndrome among Teachers in Eastern Region, Saudi Arabia

**DOI:** 10.5334/tohm.1123

**Published:** 2026-01-09

**Authors:** Zainab Saeed Mohammed Alwusaybie, Mashael Mubarak Saeed AlQuaimi, Ziyad Bandar Ali Alotaibi, Montather Akeel Nasser Alshik Ali, Walaa Mohammed Ali Alamer, Motaz Dhafer Ali Alqahtani

**Affiliations:** 1 College of Medicine, King Faisal University, Al-Ahsa, Saudi Arabia; 2 Department of Neurology, Movement Disorders Unit Prince Saud Bin Jalwi Hospital Al-Ahsa, Saudi Arabia

**Keywords:** Tourette Syndrome, Tic Disorders, Teacher Knowledge, Awareness, Saudi Arabia, Neurodevelopmental Disorders

## Abstract

**Background:**

Tourette syndrome (TS) is a neurodevelopmental disorder characterized by multiple motor and vocal tics and associated with comorbidities such as obsessive-compulsive disorder (OCD) and attention-deficit/hyperactivity disorder (ADHD). Teachers’ understanding of TS is critical for recognizing symptoms and providing effective support in the classroom.

**Methods:**

A cross-sectional study was conducted in 2024 among teachers in the Eastern Province of Saudi Arabia. A validated online questionnaire was distributed to a randomly selected sample of primary, intermediate, and high school teachers from both governmental and private schools. Knowledge was assessed using a 23-item tool and categorized as poor, moderate, or good.

**Results:**

Among the 305 participants, the majority were female (56.1%) and Saudi nationals (94.8%). A large proportion (41.3%) had substantial teaching experience (≥17 years). Overall, 57% of teachers demonstrated poor knowledge of TS, while only 5.9% showed good knowledge. Although understanding of motor and vocal tics was relatively high (65.9%), awareness of common comorbidities was lower (OCD: 29.2%; ADHD: 50.8%). Most teachers (84.9%) reported no personal experience with students with TS. A significant positive correlation was found between prior experience with TS and higher knowledge scores (p < 0.05). The primary source of information was the internet and social media (35.1%), with very few teachers citing formal training.

**Conclusion:**

A significant knowledge gap regarding TS exists among schoolteachers in Eastern Saudi Arabia. The reliance on informal sources over structured training highlights an urgent need for targeted educational programs and professional development workshops. Enhancing teacher preparedness is essential for creating inclusive learning environments that support the academic and social success of students with TS.

## Research in Context

### Evidence before this study

Tourette’s Syndrome (TS) is a complex neurodevelopmental disorder that commonly co-occurs with ADHD and OCD, potentially affecting a student’s academic performance and social development. While international studies have examined the clinical aspects of TS and teacher awareness in Western countries, there has been a lack of empirical data from Saudi Arabia, particularly in the Eastern Province. Prior local studies, such as one in Riyadh, focused primarily on medical students and physicians, not educators. No published studies have systematically evaluated the level of TS-related knowledge among schoolteachers, despite their pivotal role in identifying symptoms, implementing classroom accommodations, and reducing stigma. This study addresses a significant knowledge gap in understanding how well-prepared Saudi teachers are to support students with TS.

### Added value of this study

This is the first large-scale cross-sectional study to assess knowledge, awareness, and attitudes about Tourette’s Syndrome among teachers in the Eastern Province of Saudi Arabia. Surveying 305 teachers across various school levels and regions, the study found that 57% had poor overall knowledge of TS, while only 5.9% demonstrated good knowledge. Teachers were more familiar with motor and vocal tics (65%), but had limited understanding of associated conditions like OCD (29%) and ADHD (51%), as well as appropriate classroom interventions. The most common source of information was the internet and social media (35%), with minimal reliance on formal training programs. A statistically significant relationship was found between prior experience with TS and higher knowledge scores (p < 0.05), highlighting the importance of practical exposure in improving awareness.

### Implications of all the available evidence

The results demonstrate a pressing need for structured and targeted training programs for educators regarding TS and related neurodevelopmental disorders. Given the high percentage of teachers lacking sufficient knowledge and relying on informal sources, educational authorities should integrate evidence-based TS content into teacher training curricula, workshops, and ongoing professional development. Moreover, cross-sector collaboration between educational institutions and healthcare providers could facilitate school-based awareness campaigns and teacher support systems. Improving teacher preparedness will be key to reducing stigma, enabling effective classroom management, and fostering inclusive educational environments for students with TS across Saudi Arabia.

## Introduction

Tourette’s syndrome (TS) is a neurodevelopmental disorder that classically begins in childhood [1]. It is characterised by multiple phonic and motor tics, which can vary in complexity [1,2]. Tics are atypical movements or vocalisations [3]. The onset of tics usually occurs between the ages of four and six, with more severe tics appearing between the ages of 10 and 12 [2]. In the majority of cases, tic disorders are benign and tend to improve during adolescence, with casual improvement in about 90% of patients [4].

The prevalence of TS among children is estimated to be between 0.3% and 0.9% [2]. To diagnose TS, the Diagnostic and Statistical Manual for Mental Disorders (DSM-5) requires the presence of at least two motor tics and one vocal tic for 12 months in individuals under the age of 18 [1,5]. TS is often accompanied by other behaviour disorders, such as obsessive-compulsive disorder (OCD) and attention deficit hyperactivity disorder (ADHD). Approximately 25% to 30% of TS patients have OCD, while 50% have ADHD, which can have a significant impact on their overall clinical presentation [1]. The prevalence of ADHD in the general population is around 5%, while OCD prevalence ranges from 1.9% to 3.2% [3]. Therefore, the combined prevalence of TS, ADHD, and OCD is estimated to be approximately 10% of the general population [3].

The precise cause of TS is still not fully understood, but most research suggests that it is a genetic disorder involving abnormalities in neurotransmission at the synapses [6]. Treatment for TS and other tic disorders can involve pharmaceutical and non-pharmaceutical. However, programs that utilise positive reinforcement are particularly effective in managing tic disorders and can potentially reduce the need for medication [1]. Children and teenagers with behavioural traits associated with TS may be more susceptible to bullying and difficulties in social interaction, which could contribute to the development of psychiatric symptoms such as anxiety [7].

## Methods

### Study Design

A cross-sectional study was conducted between January 2024 and May 2024. A self-administered structured questionnaire was used to assess teachers’ knowledge and perception of TS in the Eastern Province of Saudi Arabia.

### Study Area

This study was conducted in the Eastern Province of Saudi Arabia, in both urban and rural areas such as Al-Hasa, Dammam, and Al-Khobar. These regions were selected due to their diverse demographics and the presence of both governmental and private schools, ensuring a broad representation of teachers.

### Study Population

The study targeted school teachers working in primary, intermediate, and high schools within the Eastern Province. The inclusion of a wide range of educational levels ensured a comprehensive understanding of the teaching population’s knowledge and perceptions of TS.

### Sample Size and Sampling Technique

The sample size was calculated based on the following parameters: a 95% confidence interval, a 5% margin of error, and an estimated population of teachers in the region. A final sample size of 305 participants was determined to ensure statistical power and precision. A stratified random sampling technique was employed, dividing the population into strata based on school type (governmental vs. private), gender, and teaching experience. Random selection within each stratum minimized sampling bias and ensured proportional representation.

#### Inclusion Criteria

- Teachers employed in primary, intermediate, or high schools.
- Educators in both governmental and private schools.
- Participants currently residing and working in the Eastern Province of Saudi Arabia.

#### Exclusion Criteria

- University instructors, online-only educators, and those teaching outside the Eastern Province.
- Teachers without direct classroom interaction with students.

### Data Collection Tools

Data were collected using a pre-validated, structured questionnaire administered via Google Forms. The questionnaire was developed based on a comprehensive literature review and refined through expert consultation to ensure content validity. It comprised four sections:

- **Demographics:** Age, gender, education level, teaching speciality, years of teaching experience, and personal experience with TS students.
- **Knowledge Assessment:** 23 items assessing knowledge of TS symptoms, diagnostic criteria, and comorbid conditions (e.g., OCD and ADHD).
- **Classroom Interventions:** Questions evaluating teachers’ familiarity with and confidence in implementing modifications for TS students, such as seating arrangements, test accommodations, and peer education.
- **Treatment Options:** Items assessing awareness of medical and behavioural management strategies for TS.

Knowledge was assessed using 23 items. Each correct response was scored as 1 and incorrect or ‘don’t know’ as 0, yielding a total score range of 0–23. The total score was converted to a percentage (score/23 × 100) and categorised using modified Bloom’s cut-off points: poor (<60%), moderate (60–79%), and good (80–100%) [8].

### Pilot Study

A pilot study was conducted with 30 randomly selected teachers to evaluate the reliability and clarity of the questionnaire. Cronbach’s alpha was calculated to assess internal consistency, yielding a value of 0.87, indicating high reliability. Feedback from participants was incorporated to refine ambiguous questions, ensuring the instrument’s validity and feasibility for large-scale application.

### Statistical Analysis

All statistical analyses were performed using IBM SPSS Statistics (Version 27.0). Descriptive statistics, including frequencies, percentages, means, and standard deviations, were used to summarize the demographic characteristics of the participants and their knowledge scores. Inferential analyses were conducted to examine relationships and differences among variables. Associations between categorical variables, such as knowledge levels and demographic characteristics, were evaluated using Chi-square tests. Comparisons of mean knowledge scores across different demographic groups were performed using independent t-tests and one-way Analysis of Variance (ANOVA). A p-value of less than 0.05 was considered statistically significant for all tests. Furthermore, a post-hoc power analysis was conducted, confirming that the achieved sample size provided 80% power to detect meaningful differences at a 5% significance level, thereby ensuring the robustness of the statistical interpretations.

### Ethical Considerations

Ethical approval was obtained from the King Faisal University Research Ethics Committee (IRB Approval: ETHICS182). All participants provided informed consent electronically before completing the questionnaire. Data confidentiality and anonymity were strictly maintained throughout the study, and participants were informed of their right to withdraw at any stage without consequences. The study adhered to the ethical principles outlined in the Declaration of Helsinki.

## Results

### Baseline Characteristics of Participants

The sample consisted of 43.93% males (n = 134) and 56.07% females (n = 171). Most participants were Saudi (94.75%, n = 289), with 93.75% (n = 285) holding a bachelor’s degree. The majority of teachers specialised in mathematics (19.34%, n = 59), science (24.59%, n = 75), or English language (13.77%, n = 42).

Regarding teaching experience, 41.31% (n = 126) had 17+ years, and 64.26% (n = 196) worked in large districts (more than 300 students). Participants taught across various grade levels: elementary (36.39%, n = 111), junior high (21.64%, n = 66), and high school (37.38%, n = 114). In terms of knowledge of TS, 64.26% (n = 196) had little or no knowledge. Most learned about the disorder through the internet (35.08%, n = 107), and 84.92% (n = 259) had no personal experience with children with TS. Regarding intervention techniques, 47.90% (n = 146) felt competent in parent consultation, and 60.30% (n = 184) in teacher education (Table 1).

**Table 1:** Demographic characteristics and background information among the studied sample (N = 305).


CHARACTER	COUNT	%

**Gender**		

Male	134	43.93

Female	171	56.07

**Nationality**		

Saudi	289	94.75

Non-Saudi	16	5.25

**Level of education**		

Bachelor Degree	285	93.75

Master’s Degree	12	3.95

Doctorate	3	0.99

Education Specialist	4	1.32

**Teaching specialty**		

Mathematics	59	19.34

Science	75	24.59

English language	42	13.77

Arabic language	39	12.79

Physical education	7	2.30

Arts	2	0.66

Computer science	10	3.28

Social studies	20	6.56

Islamic studies	24	7.87

Management science	9	2.95

Life skills	8	2.62

Preschool	2	0.66

Special education	8	2.62

**Years of employment as a school teacher**		

0 to 6	72	23.61

7 to 12	64	20.98

12 to 16	43	14.10

17 or more	126	41.31

**Size of the district**		

0–100 students	26	8.52

101–200 students	42	13.77

201–300 students	41	13.44

More than 300	196	64.26

**Grade levels of working**		

Preschool	14	4.59

Elementary	111	36.39

Junior high	66	21.64

High school	114	37.38

**Working regain in Eastern**		

Alhasa	182	59.87

Aljubail	3	0.99

Al khobar	11	3.62

Alkhafji	1	0.33

Al dammam	61	20.07

Dahran	1	0.33

Qatif	28	9.21

Hafar al batin	17	5.59

**Knowledge rate level about Tourette’s syndrome**		

Little or no knowledge	196	64.26

Somewhat knowledgeable	98	32.13

Very knowledgeable	11	3.61

**Sources that primarily learn about the disorder**		

University or College training program	24	7.87

Workshops or seminars	6	1.97

Independent study	10	3.28

Parents of children with the disorder	13	4.36

Internet and social media	107	35.08

**Personal experience working with children with Tourette Syndrome or other movement disorders**		

Yes	46	15.08

No	259	84.92

**Intervention techniques to feel competent to employ with regard to Tourette Syndrome**		

Parent consultation	146	47.9

Teacher consultation	82	26.9

Parent education	172	56.4

Teacher education	184	60.3

Individual counseling	81	26.6

Social/group counselling	119	39.0

None of these	45	14.8

Don’t know	10	2.6


### General Knowledge about Tourette’s Syndrome

A majority of teachers correctly identified that both motor and vocal tics are associated with TS (65.90%, n = 201). However, 40.98% (n = 125) were unsure about the ability to suppress tics for varying lengths of time, and 46.56% (n = 142) were unsure of the onset of symptoms before age 18. Regarding the need for symptoms to be present continuously for one year, 43.61% (n = 133) did not know, while 24.26% (n = 74) believed the statement to be false. Teachers’ views on the nature of TS included 46.56% (n = 142) agreeing that it is a neurological and genetic disorder. However, 39.02% (n = 119) did not know that children with Tourette’s often have obsessive-compulsive disorder, and 40.66% (n = 124) believed that stimulant medications were most effective for treatment. A majority (50.82%, n = 155) agreed that children with TS often have ADHD (Table 2).

**Table 2:** The elements of general knowledge of Tourette’s syndrome among teachers in the eastern region.


STATEMENTS	RESPONSES n(%)		

	TRUE	FALSE	DON’T KNOW

Both motor (physical) tics and (vocal) phonic tics	201(65.90)	22(7.21)	82(26.89)

Ability to suppress tics for varying lengths of time	84(27.54)	96(31.48)	125(40.98)

Both inappropriate words and physical restlessness	185(60.66)	36(11.80)	84(27.54)

Onset of symptoms before the age of 18	142(46.56)	54(17.70)	109(35.74)

Symptoms have to be present continuously for 1 year without “symptom-free” periods.	98(32.13)	74(24.26)	133(43.61)

	**AGREE**	**DISAGREE**	**DON’T KNOW**

Tourette Syndrome is a neurological and genetic disorder	142(46.56)	72(23.61)	91(29.84)

Children with Tourette Syndrome often have obsessive-compulsive disorder	89(29.18)	97(31.80)	119(39.02)

Tourette Syndrome is most effectively treated with stimulant medications	87(28.52)	94(30.82)	124(40.66)

Tourette Syndrome occurs in equal numbers of boys and girls	104(34.10)	93(30.49)	108(35.41)

Children with Tourette Syndrome often have attention deficit hyperactivity disorder	155(50.82)	48(15.74)	102(33.44)

Saying inappropriate words is the most common symptom of Tourette Syndrome	129(42.30)	73(23.93)	103(33.77)

The symptoms of Tourette Syndrome may not be seen for weeks at a time	132(43.28)	56(18.36)	117(38.36)

Look for learning problems in math and written language, and reading in a child with Tourette’s syndrome.	144(47.21)	61(20)	100(32.79)


**Figure 1:**
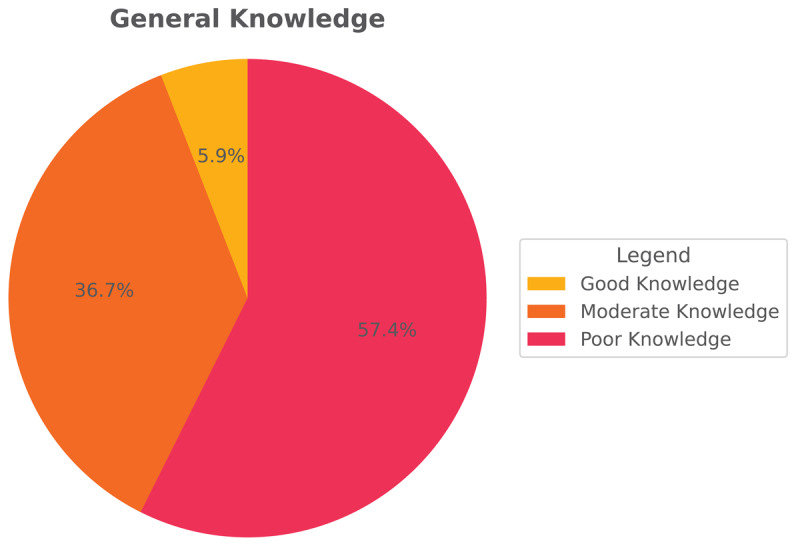
Categories of general knowledge of Tourette’s syndrome among teachers in the eastern region.

**Figure 2:**
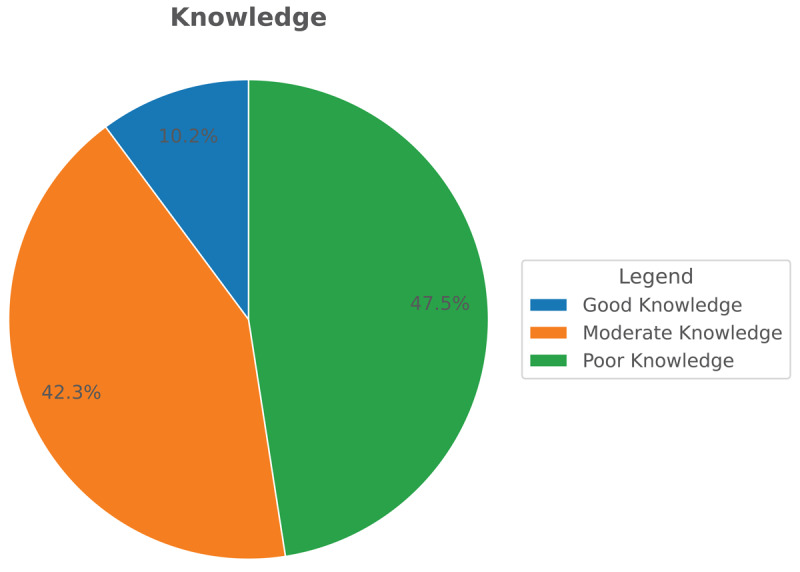
Flow chart of the final included studies in the review based on the PRISMA statement.

Figure 1 shows the general knowledge of TS among teachers in the eastern region. 57% of them have poor knowledge, and a small number by 5.9% have good knowledge Figure 2 shows the knowledge about symptoms and treatment of TS among teachers in the eastern region. 47% of them have poor knowledge, and a small number, by 10% have good knowledge.

### Knowledge about Appropriate Response to Children with Tourette’s Syndrome

In Table 3, teachers showed varying levels of knowledge regarding appropriate responses to children with TS. A significant proportion of teachers (42.30%, n = 129) deemed it inappropriate to allow a child to periodically leave the classroom, while 20.98% (n = 64) thought it was appropriate. Regarding the establishment of a separate test-taking room, 27.54% (n = 84) found it appropriate, and 14.75% (n = 45) considered it inappropriate. Most teachers (53.77%, n = 164) agreed that educating classmates about TS is appropriate, while 13.77% (n = 42) considered it inappropriate.

**Table 3:** The elements assess the knowledge of symptoms and treatment of Tourette’s syndrome among teachers in the eastern region.


STATEMENTS	RESPONSES n(%)				

	INAPPROPRIATE	SOMETIMES	OFTEN	APPROPRIATE	DON’T KNOW

Allow the child to periodically leave the classroom	64(98.20)	129(30.42)	38(46.12)	30(84.9)	44(43.14)

Establish a separate test-taking room for the child	45(75.14)	76(92.24)	57(69.18)	84(54.27)	43(10.14)

Educate the child’s classmates about Tourette Syndrome	26(52.8)	34(15.11)	42(77.13)	164(77.53)	39(79.12)

Gently remind the child to try and control his/her tics	46(08.15)	61(20)	67(97.21)	90(51.29)	41(44.13)

Seat the child near the front of the classroom	45(75.14)	65(31.21)	41(44.13)	110(07.36)	44(43.14)

Establish time limits for assignments and tests	53(38.17)	62(33.20)	52(05.17)	92(16.30)	46(08.15)

	**AGREE**		**DISAGREE**		**DON’T KNOW**

A child with Tourette Syndrome sometimes needs no treatment	138(25.45)		83(54.27)		84(21.27)

Drugs can sometimes be used for children with tourette syndrome	180(02.59)		37(13.12)		88(85.28)

Surgery is not needed for children with Tourette Syndrome	132(28.43)		57(69.18)		116(03.38)

All children with Tourette Syndrome will get better as they get older	138(25.45)		54(70.17)		113(05.37)


In terms of classroom management, 29.51% (n = 90) agreed with gently reminding a child to control their tics, while 29.51% (n = 90) deemed it appropriate to seat the child near the front of the classroom. Time limits for assignments and tests were viewed as appropriate by 30.16% (n = 92), with 17.38% (n = 53) finding them inappropriate.

Concerning treatment, 45.25% (n = 138) disagreed with the idea that children with TS sometimes need no treatment, while 59.02% (n = 180) agreed that drugs can sometimes be used. Teachers largely agreed that surgery is not needed for children with Tourette’s (43.28%, n = 132), while 45.25% (n = 138) disagreed with the belief that all children with Tourette’s will improve as they age.

### Factors Influencing Participants’ Knowledge

Table 4 assesses the overall knowledge categorized with regard to related factors in the study. By using the Chi-Square test and Wilcoxon sample t-test, the table shows there are differences between knowledge categories and the previous experience of working with children with the disease or with other movement disabilities. This difference is statistically significant.

**Table 4:** Associate the categories of total general knowledge about Tourette’s syndrome with related factors.


FACTORS	KNOWLEDGE CATEGORIZES n(%)			P-VALUE

	GOOD	MODERATE	POOR	

**Gender**				

Male	7(22.5)	44(84.32)	83(94.61)	0.3

Female	11(43.6)	68(77.39)	92(80.53)	

***Age***	45(38–51)	45(37–48)	41(32–48)	0.07

**Teaching specialty**				

Mathematics	3(08.5)	17(81.28)	39(10.66)	0.4

Science	4(33.5)	32(67.42)	39(52)	

English language	2(76.4)	10(81.23)	30(43.71)	

Arabic language	3(69.7)	16(03.41)	20(28.51)	

Physical education	0	2(57.28)	5(43.71)	

Arts	0	0	2(100)	

Computer science	1(10)	5(50)	4(40)	

Social studies	2(10)	12(60)	6(30)	

Islamic studies	2(33.8)	10(67.41)	12(50)	

Management science	1(11.11)	1(11.11)	7(78.77)	

Life skills	0	3(50.37)	5(50.62)	

Preschool	0	1(50)	1(50)	

Special education	0	3(50.37)	5(50.62)	

**Personal experience working with children with Tourette Syndrome or other movement disorders**				0.001**

Yes	4(8.70)	27(58.70)	15(32.61)	

No	14(41.5)	85(82.32)	160(78.61)	


Table 5 assesses the knowledge of symptoms and treatment of TS, categorized according to related factors in the study. Using the Chi-Square test and Wilcoxon sample t-test, the table shows differences between knowledge categories and previous experience working with children with the disease or with other movement disabilities. This difference is statistically significant.

**Table 5:** Associate the categories of total knowledge of symptoms and treatment of Tourette’s syndrome with related factors.


FACTORS	KNOWLEDGE CATEGORIZES n(%)			P-VALUE

	GOOD	MODERATE	POOR	

**Gender**				0.3

Male	12(8.96)	52(38.81)	70(52.24)	

Female	19(11.11)	77(45.03)	75(43.86)	

***Age***	45(38–51)	45(37–48)	41(32–48)	0.07

**Teaching specialty**				

Mathematics	6(10.17)	19(32.20)	34(57.63)	0.2

Science	2(2.67)	34(45.33)	39(52)	

English language	6(14.29)	18(42.86)	18(42.86)	

Arabic language	4(10.26)	18(46.15)	17(43.59)	

Physical education	0	2(28.57)	5(71.43)	

Arts	0	1(50)	1(50)	

Computer science	2(20)	6(60)	2(20)	

Social studies	3(15)	9(45)	8(40)	

Islamic studies	5(20.83)	13(54.17)	6(25)	

Management science	2(22.22)	2(22.22)	5(55.56)	

Life skills	0	3(37.50)	5(62.50)	

Preschool	0	1(50)	1(50)	

Special education	1(12.50)	3(37.50)	4(50)	

**Personal experience working with children with Tourette Syndrome or other movement disorders**				

Yes	6(13.04)	29(63.04)	11(23.91)	0.002**

No	25(9.65)	100(38.61)	134(51.74)	


### Summary of Results

The demographic profile of the 305 participating teachers revealed a predominance of female Saudi nationals holding a bachelor’s degree, with specializations primarily in mathematics, science, and English. Teaching experience varied widely, with a substantial proportion working in large school districts and across elementary, junior high, and high school levels.

Assessment of TS-related knowledge identified significant gaps among educators. The majority of teachers reported little to no prior knowledge of TS, with most indicating the internet as their primary information source. While a majority correctly identified the presence of both motor and vocal tics as core symptoms of TS, understanding of other critical aspects was limited. Notably, teachers demonstrated uncertainty regarding the suppressibility of tics, the diagnostic requirement of symptom persistence for one year, and the frequent co-occurrence of obsessive-compulsive disorder (OCD). However, approximately half of the participants recognized the association between TS and attention-deficit/hyperactivity disorder (ADHD). Overall, 57% of teachers were categorized as having poor general knowledge of TS, with only 5.9% demonstrating good knowledge.

Regarding classroom management and intervention strategies, perspectives were mixed. Most teachers supported educating classmates about TS to foster an inclusive environment. However, there was considerable division and uncertainty about practical accommodations, such as allowing periodic classroom exits, providing separate testing environments, or modifying seating arrangements. In terms of treatment, a common misconception was that stimulant medications are the most effective approach for TS.

Finally, analysis of influencing factors revealed that prior personal or professional experience with students with TS or other movement disorders was significantly associated with higher knowledge scores. This correlation underscores the value of direct exposure in improving awareness and understanding. The findings collectively address the study’s core aim of evaluating the current state of awareness, attitudes, and perceptions of TS among schoolteachers in the region.

The study found a statistically significant correlation between previous experience with children with TS or other movement disorders and higher knowledge scores (p < 0.05). In conclusion, the study highlighted a lack of adequate knowledge about TS among teachers in the Eastern region. Limited exposure and training were significant contributing factors.

## Discussion

This study assessed the knowledge, perceptions, and attitudes of teachers in the Eastern region of Saudi Arabia regarding TS. This study aims to address gaps in awareness and its implications for classroom strategies and student outcomes.

The demographic data revealed that most participants were female Saudi nationals with a bachelor’s degree, primarily teaching subjects like mathematics, science, and English. These findings are consistent with the general representation of educators in the region. Most teachers had significant teaching experience working in large districts. So, their exposure to TS was limited. As results show, 64.26% of teachers reported little or no knowledge about TS. The majority rely on the Internet as the primary source of information for TS. This indicates a lack of structured and reliable training programs. As previously highlighted in studies on other neurological disorders in Saudi Arabia [9].

Many teachers successfully identified motor and vocal tics as characteristic symptoms of TS. However, significant gaps in knowledge were shown regarding other critical aspects, like tic suppression, symptom onset, and treatment necessity. A total of 40.98% were unsure about tic suppression, and 45.25% disagreed that children with TS might not always require treatment. These findings are consistent with the global research conducted by Ueda et al. (2021), which discusses the correlates and clinical implications of tic suppressibility [10]. Their study paper highlights that tic suppressibility is a key feature of tic disorders. Many children were able to suppress tics for varying periods. However, it also notes that understanding tic suppression and its implications is important for effective treatment. The study emphasizes that not all children with TS require treatment, as some may manage their symptoms effectively [10,11]. This shows the need for improved education among teachers regarding tic disorders and their management.

Teachers also show mixed perspectives on effective classroom strategies for students with TS. While many supported educating classmates about the disorder (53.77%). Some were divided on interventions like periodic classroom exits (42.30% deemed it inappropriate) and test accommodations. These findings suggest an inconsistency in understanding the management of TS in educational settings. It is impacting students’ academic experiences and social integration. Previous studies have shown that teacher training in neurological disorders significantly improves classroom strategies and student outcomes [12].

Another study also highlights that educators should receive training on TS to understand its complexities and impact on learning. This includes recognizing tics and triggers [13]. Providing information to classmates about TS can create a supportive environment, reduce stigma, and encourage empathy [14]. Another study also showed that classroom management for TS involves ignoring tics, allowing breaks, and modifying tasks [15]. Teachers should adopt strategies to meet individual needs. Teachers also should promote peer education for acceptance and focus on quality over quantity in assignments to support students. Elsom and Hansen (2021) suggest teachers should adopt strategies and the learning environment based on the principles of universal design [13]. It will effectively support students with TS in higher education settings.

A statistically significant association was observed between prior experience with students with TS or other movement disorders and higher knowledge scores (p < 0.05). Teachers with firsthand experience showed better understanding and were more likely to adopt effective intervention strategies. This finding aligns with (Holly, 2009), who reported that teachers with firsthand experience of TS are more likely to understand the disorder and implement effective intervention strategies. Their awareness and knowledge significantly enhance their ability to support students with TS academically and socially. Experienced teachers are more likely to implement evidence-based interventions, such as behavioral strategies and classroom accommodations, which can significantly improve academic performance and social adjustment for students with TS [15].

The study underscores the importance of structured teacher training programmes on TS and related neurodevelopmental disorders. It recommends that teacher preparation courses incorporate dedicated modules on such conditions, ensuring educators enter the profession equipped with foundational knowledge to support pupils with diverse needs. In addition, workshops, seminars, and school-based initiatives can be instrumental in bridging knowledge gaps and challenging common misconceptions. Collaborative efforts between educational institutions and healthcare organizations are also vital in developing awareness campaigns aimed at reducing stigma and fostering inclusivity within school environments. This study offers valuable insights; however, it is not without limitations. The reliance on self-reported data may introduce bias, and the cross-sectional design restricts the ability to draw causal inferences. An additional limitation is the variability in teachers’ educational and scientific backgrounds, which may influence baseline knowledge levels. Furthermore, sources of information were self-reported and broadly categorised (e.g., internet or social media) without verification or differentiation between certified educational programs and informal content. Future studies should further classify training sources and assess the impact of structured educational interventions. Finally, the focus on the Eastern Province may constrain the generalizability of the findings to wider populations.

## Conclusion

This study identified an important lack of knowledge and awareness about TS among teachers in the Eastern Province of Saudi Arabia. While teachers demonstrated some understanding of specific aspects, such as motor and vocal tics. Their overall awareness of TS symptoms, comorbidities, and effective classroom strategies was limited. Most participants relied on informal sources, such as social media and the internet, to learn about TS. So, it is highlighting the absence of structured and formal training opportunities in this area.

The findings suggest that prior exposure to students with TS positively impacts teachers’ knowledge levels, highlighting the importance of experience-based learning. However, the lack of professional development programs and teacher education initiatives targeting TS remains a critical challenge. Addressing this gap is essential to creating a supportive and inclusive environment for students with TS, enabling them to achieve their full academic potential.
